# Fish oil supplements, oxidative status, and compliance behaviour: Regulatory challenges and opportunities

**DOI:** 10.1371/journal.pone.0244688

**Published:** 2020-12-31

**Authors:** Ammar Abdulrahman Jairoun, Moyad Shahwan, Sa’ed H. Zyoud

**Affiliations:** 1 Health and Safety Department, Dubai Municipality, Dubai, UAE; 2 Discipline of Social and Administrative Pharmacy, School of Pharmaceutical Sciences, Universiti Sains Malaysia, Pulau Pinang, Malaysia; 3 Department of Clinical Sciences, College of Pharmacy and Health Sciences, Ajman University, Ajman, UAE; 4 Center of Medical and Bio-allied Health Sciences Research, Ajman University, Ajman, UAE; 5 Poison Control and Drug Information Center (PCDIC), College of Medicine and Health Sciences, An-Najah National University, Nablus, Palestine; 6 Clinical Research Centre, An-Najah National University Hospital, Nablus, Palestine; Michigan State University, UNITED STATES

## Abstract

**Background:**

Fish oil supplements that are rich in omega-3 long-chain polyunsaturated fatty acids (n-3 PUFAs). PUFAs are among the most widely-used dietary supplements globally, and millions of people consume them regularly. There have always been public concerns that these products should be guaranteed to be safe and of good quality, especially as these types of fish oil supplements are extremely susceptible to oxidative degradation.

**Objectives:**

The aim of the current study is to investigate and examine the oxidation status of dietary supplements containing fish oils and to identify important factors related to the oxidation status of such supplements available in the United Arab Emirates (UAE).

**Methods:**

A total of 44 fish oil supplements were analysed in this study. For each product, the oxidative parameters peroxide value (PV), anisidine value (AV), and total oxidation (TOTOX) were calculated, and comparisons were made with the guidelines supplied by the Global Organization for EPA and DHA Omega-3s (GOED). Median values for each of the above oxidative parameters were tested using the Kruskal-Wallis and Mann-Whitney U tests. P values < 0.05 were chosen as the statistically significant boundary.

**Results:**

The estimate for the average PV value was 6.4 with a 95% confidence interval (CI) [4.2–8.7] compared to the maximum allowable limit of 5 meq/kg. The estimate for the average P-AV was 11 with a 95% CI [7.8–14.2] compared to the maximum allowable limit of 20. The estimate for the average TOTOX value was 23.8 meq/kg with a 95% CI [17.4–30.3] compared to the maximum allowable limit of 26 according to the GOED standards.

**Conclusion:**

This research shows that most, although not all, of the fish oil supplements tested are compliant with the GOED oxidative quality standards. Nevertheless, it is clear that there should be a high level of inspection and control regarding authenticity, purity, quality, and safety in the processes of production and supply of dietary supplements containing fish oils.

## Introduction

Because dietary supplements (DSs) can become contaminated with a quantity of toxic material when they are being manufactured, transported, and stored, both regulators and the public are concerned with ensuring that such products remain safe and of good quality for consumption, especially in the long term. Globally, fish oils are one of the most popular dietary supplements available. In the United States, of the 17.7% of adults who take dietary supplements, over one-third take fish oil dietary supplements [1,2]. There are notable quantities of omega-3 long-chain polyunsaturated fatty acids (n-3 PUFAs) in fish oils, with the metabolically-active compounds being eicosapentaenoic acid (EPA) and docosahexaenoic acid (DHA) [3]. Many different benefits have been claimed for n-3 fish oils, including preventing CVD [4], reducing cognitive decline [5], and helping improve management of inflammatory diseases such as asthma, IBD, and arthritis [6]. It is suggested that having a diet that is deficient in these elements, and therefore tissues that have low levels of them, makes individuals more susceptible to chronic diseases [7]. However, because they have many double bonds in the fatty-acid chain, certain n-3 PUFAs are very susceptible to oxidation [8,9].

When fish oils oxidize, the amount of unoxidized fatty acid decreases, to be replaced by a complex mixture of secondary oxidation products (aldehydes and ketones) and liquid peroxides [8]. When antioxidants are added to fish oils, oxidation is reduced but not prevented [10]. A complex oxidation process occurs for n-3 PUFAs, and the amount and speed of fish oil oxidization can be affected by numerous elements, including the composition of fatty acids, how much O2 and light exposure the product experiences, antioxidant content, temperature, and the amount of water and heavy metals present [8].

Although it is problematic to measure particular oxidation species, by measuring peroxide values (PVs) and anisidine values (AVs), we can determine the degree of oxidation. PV indicates the primary oxidation products (lipid peroxides), and AV indicates the secondary oxidation products (aldehydes/ketones). In combination, we can use these parameters to estimate the total oxidation value (TOTOX). Several organizations have set recommended maximum levels for such indices [11,12], but such industry standards relate to palatability, with too few data available to mandate standards relating to their effect on health [13].

Pharmacopeial monographs and regional regulatory bodies have specified the pharmaceutical ingredients and the quality of DHA- and EPA-containing oils used in fish oil dietary supplements. Quality control generally covers fatty-acid content, oxidation states, environmental contaminants, and the means of measuring these contaminants in a number of different classes of marine oils. The Global Organization for EPA and DHA Omega-3s (GOED) composed the most recent report; it is an association of manufacturers producing omega-3 LCPUFA-containing products and those in related industries. GOED members must produce omega-3 LCPUFA-rich oils compliant with the limits related to primary oxidization (PV < 5 meq O2/kg), secondary oxidation (P-AV < 20), and a combination of measurements that encompasses levels of primary and secondary oxidation (TOTOX < 26) [14].

A number of studies have recently examined fatty acid content and oxidative quality for both liquid and encapsulated formulations of EPA and DHA. Whilst certain studies concluded that the majority of products tested complied with regulations [15–17], others found that a notable percentage of products tested fell short in one or more quality parameters [18–27]. As the dietary supplements market has become increasingly globalized and profitable, increasing levels of dietary supplements are now available to consumers in the United Arab Emirates (UAE), and people have ready access to unfamiliar supplements that can cause unexpected adverse effects. This has raised concerns among members of the healthcare community about the inherent safety of dietary supplements [28–31]. As yet, no UAE-based studies have explored these matters. This research gap inspired this investigation examining the oxidation status of dietary supplements containing fish oils, which also intends to identify the important factors that are related to the oxidation status of such supplements available in the UAE.

## Materials and methods

### Sample collection (sampling method)

Local business directories were searched to identify outlets selling fish oil supplements; these directories hold information on all of the UAE's pharmacies, para-pharmacies, and healthcare and nutrition retailers. In all, 1650 were found, and an Excel spreadsheet was used to create a sampling framework holding all the necessary information, including addresses, phone numbers, emails, and business names. We then created a study sample by employing basic random-sample selection based on business ID numbers, with stratification applied based on type and location. In each chosen location, random selection was made of one package of all the fish oil supplements available designed for oral use, regardless of where they had been manufactured. Every item was provided with a code reference number to allow for tracking and to obviate duplication. Details were recorded for every sample as follows: product name, brand name, item category, country of origin/manufacturer, subcategory, dosage form, batch number, barcode, size/volume, recommended dose, and the section of the shop from which the product was picked. If more than one outlet carried identical products (same name, manufacturer, formulation, barcode, and size/volume), whichever product was picked first was tested with the other products being returned. If any products shared a name but were manufactured by different companies or were in multiple formats (e.g., both capsules and tablets), they were regarded as separate products and both were tested. All the products picked were sent to a laboratory to be analysed on the day they were collected, and all the chemicals used were of analytical grade and purchased from Sigma-Aldrich USA.

### Sample preparation/testing

The European pharmacopeia standard was used to measure PV [32], employing visual titration of iodine. A volumetric flask was used for weighing 2.5 g of oil, to which 50 mL of 352 (glacial acetic acid: trimethylpentane) was added along with 500 mL of saturated potassium iodide solution. After occlusion of the flask, it was shaken vigorously for 1 minute, then 30 mL of water was added. This yellow solution underwent titration with a 0.01 N sodium thiosulfate solution until it was virtually colourless, then 500 mL of 1% starch solution was added. Once again this solution was titrated with a 0.01 N sodium thiosulfate solution until colourless. The PV in meq/l was calculated using a sodium thiosulfate solution with the formula “PV = [10 X (V—Vcontrol)]/m,” with m being the mass of the oil that had been measured earlier. Based on triplicate measurements, the intra-assay coefficient of variation was 2.6%, and the inter-assay coefficient of variation was 1.8%. The European pharmacopeia standard was used to measure AV [32], employing absorbance spectrophotometry following reaction with p-anisidine. To summarize the process briefly, a small vial was employed to measure 0.2 g of oil, to which was then added 10 mL of trimethylpentane. When measured against a reference solution of trimethylpentane, A1 absorbance was measured at 350 nm using UV-Vis-NIR Spectrophotometer UV-3600 Plus- Shimadzu. A total of 1 mL of 2.5 g/L p-anisidine was added to 5 mL of oil-trimethylpentane solution, and 10 minutes later, we measured the A2 absorbance compared to a reference solution of 5 ml trimethylpentane with 1 mL of p-anisidine in acetic acid. These absorbances were used to calculate the AV and the previously-obtained oil mass (m): “10 X [1.2 X (A2-A1)]/m.” Based on triple measures, the intra-assay coefficient of variation was 3.9%, and the inter-assay coefficient of variation was 3.8%. We then calculated TOTOX employing the formula “(2 X PV) + AV” [12]. Oxidation markers were then compared against the GOED guidelines. These guidelines have maximum recommended limits of PV 5 mEq/kg, AV 20, and TOTOX 26 [33].

### Ethical considerations

The study was approved by Institutional Review Board of An-Najah National University, reference number (Phd/2/20/15).

### Statistical analysis

Data analysis was undertaken employing SPSS version 24, Chicago, IL, US. Qualitative variables were summarized using frequencies and percentages. With each product, oxidative parameters (PV, AV, and TOTOX) were calculated and comparisons were made with the guidelines supplied by the GOED. Median values for each of the above oxidative parameters were tested using the Kruskal-Wallis and Mann-Whitney U tests. P values < 0.05 were chosen as the statistically significant boundary.

## Results

### Sample description

A total of 44 fish oil supplements were analysed in this study. Of the 44 samples, 9 (20.5%) were manufactured in the USA, 17 (38.6%) in the EU, 6 (13.6%) in India and 12 (27.3%) in Canada. Regarding the packaging type of the fish oil supplements, 40 (90.9%) were plastic and 4 (9.1%) were glass. The packaging colours of the tested samples were as follows: transparent (56.8%), aluminium strips (22.7%) and amber coloured (20.5%). Of the samples, syrup constituted 11.4%, soft gels 31.8%, chewables 6.8% and capsules 50.0%. Of the total samples, 15 (34.1%) contained vitamin E and 29 (65.9%) did not contain vitamin E (Table 1).

**Table 1:** Number and percentages of fish oil supplement characteristics.

Characteristic	Groups	Frequency	Percentage
Country of origin	USA	9	20.5%
	EU	17	38.6%
	India	6	13.6%
	Canada	12	27.3%
Packaging type	Plastic	40	90.9%
	Glass	4	9.1%
Packaging colour	Transparent	25	56.8%
	Aluminium strips	10	22.7%
	Amber coloured	9	20.5%
Dosage forms	Syrup	5	11.4%
	Soft gel	14	31.8%
	Chewable	3	6.8%
	Capsule	22	50.0%
Presence of vitamin E	Yes	15	34.1%
	No	29	65.9%

### Assessment of oxidation status of fish oil supplements

Estimates of the mean concentration with confidence interval (CI) and standard deviation for oxidation parameters (PV, P-AV, and TOTOX) of the fish oil supplements are presented in Table 2.

**Table 2:** Estimates of concentration of oxidation parameters from fish oil supplements and maximum allowable limit (n = 44).

Oxidation parameters	Maximum allowable limit	LOD	Products exceeding maximum limit		Estimates of concentration					
			N	%						
					Mean	± SD	95% CI		Median	Q3-Q1
Peroxide value	< 5 meq O2/kg	0.1 meq O2 / Kg	18	40.9%	6.4	7.4	4.2	8.7	3.9	7.7–2.1
Anisidine value	< 20	1	3	6.8%	11	10.5	7.8	14.2	9	13.5–5
TOTOX value	< 26	0.1	12	27.3%	23.8	21.2	17.4	30.3	17.4	29–13

Maximum allowable limits according to the GOED. LOD: Limit of detection according to Emirates International Accreditation Centre (EIAC).

**Fig 1:**
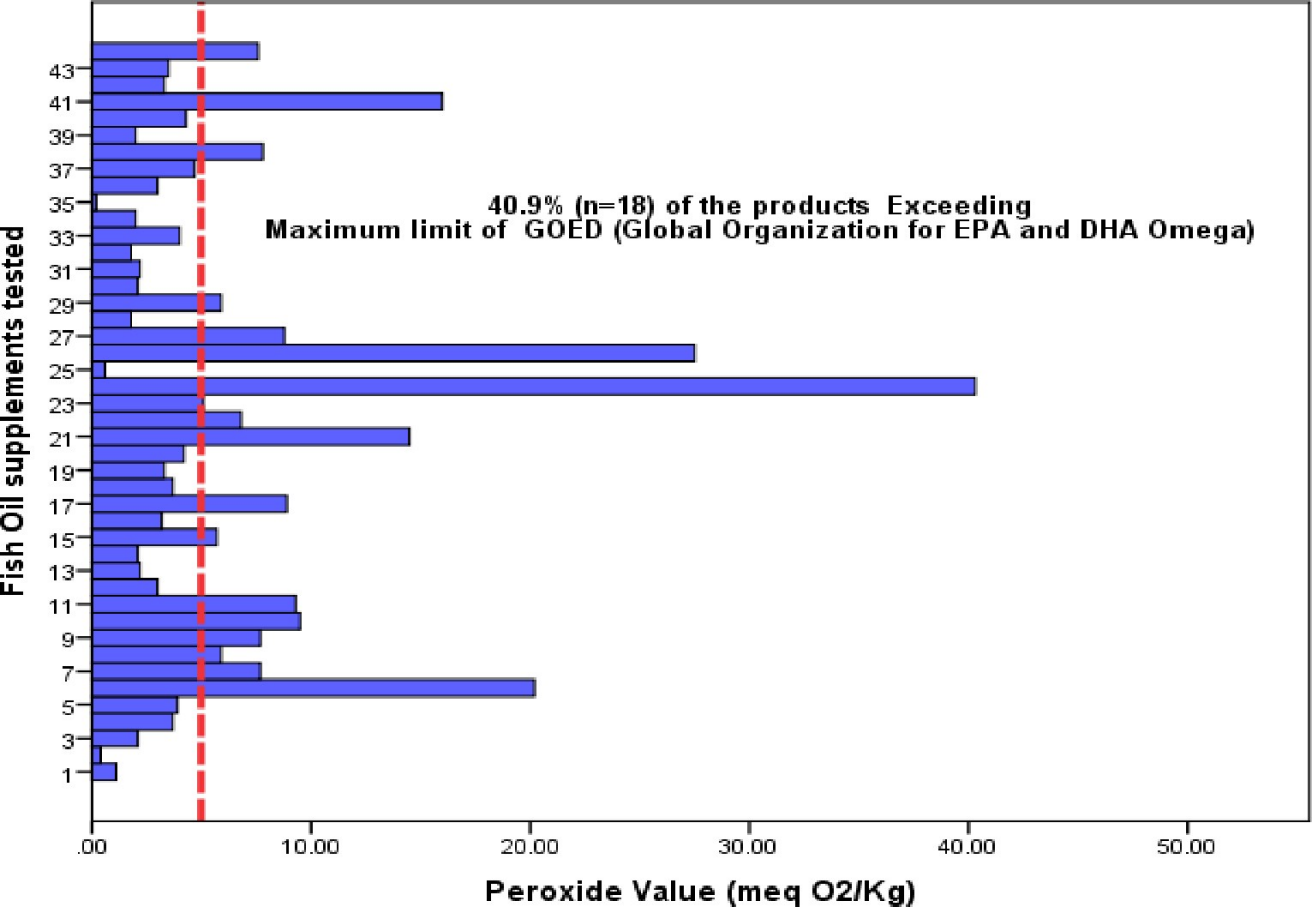
Bar chart of the estimated PV (meq/kg) for fish oil supplements (n = 44). The vertical dashed line is the maximum allowable limit of the TOTOX.

**Fig 2:**
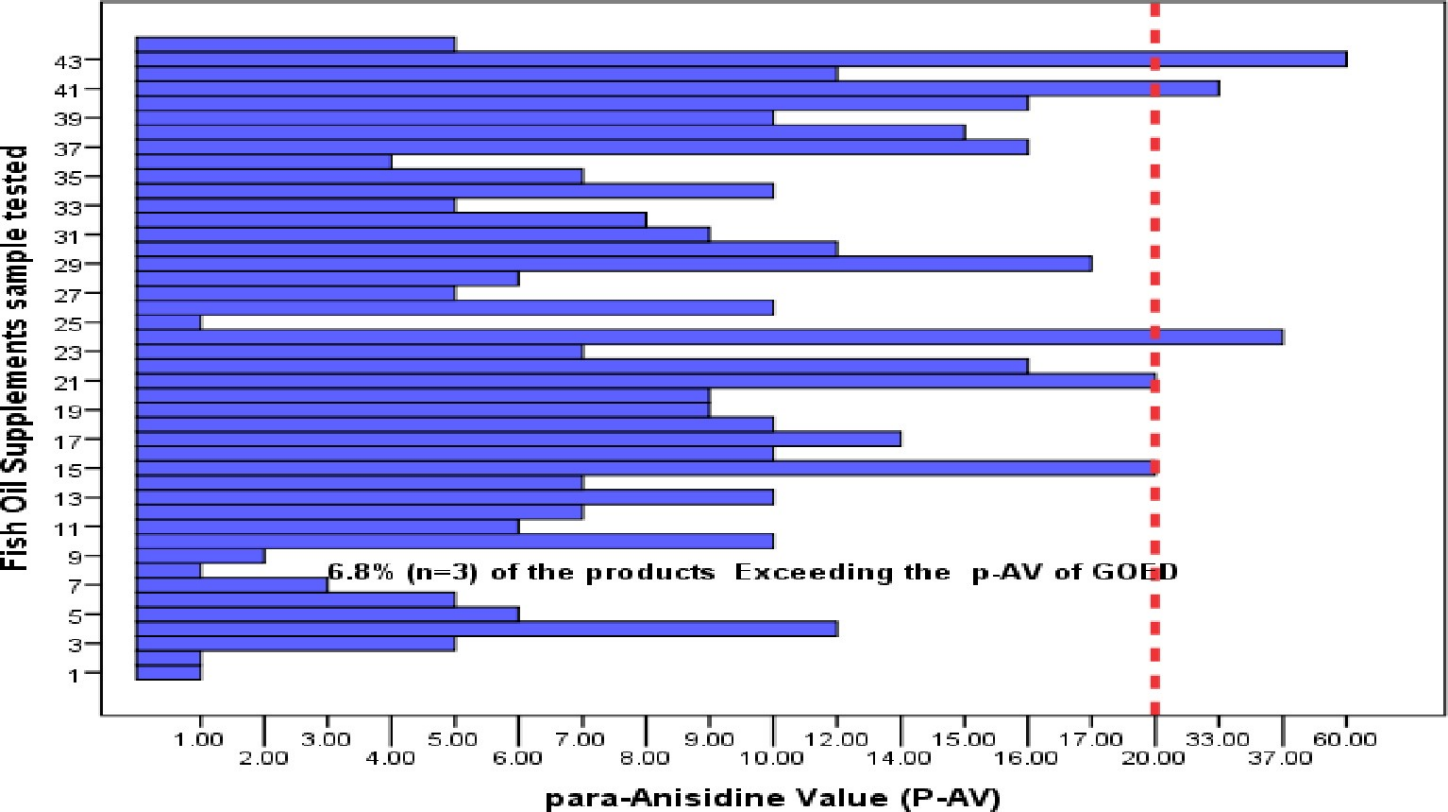
Bar chart of the estimated P-AV for fish oil supplements (n = 44). The vertical dashed line is the maximum allowable limit of the P-AV.

**Fig 3:**
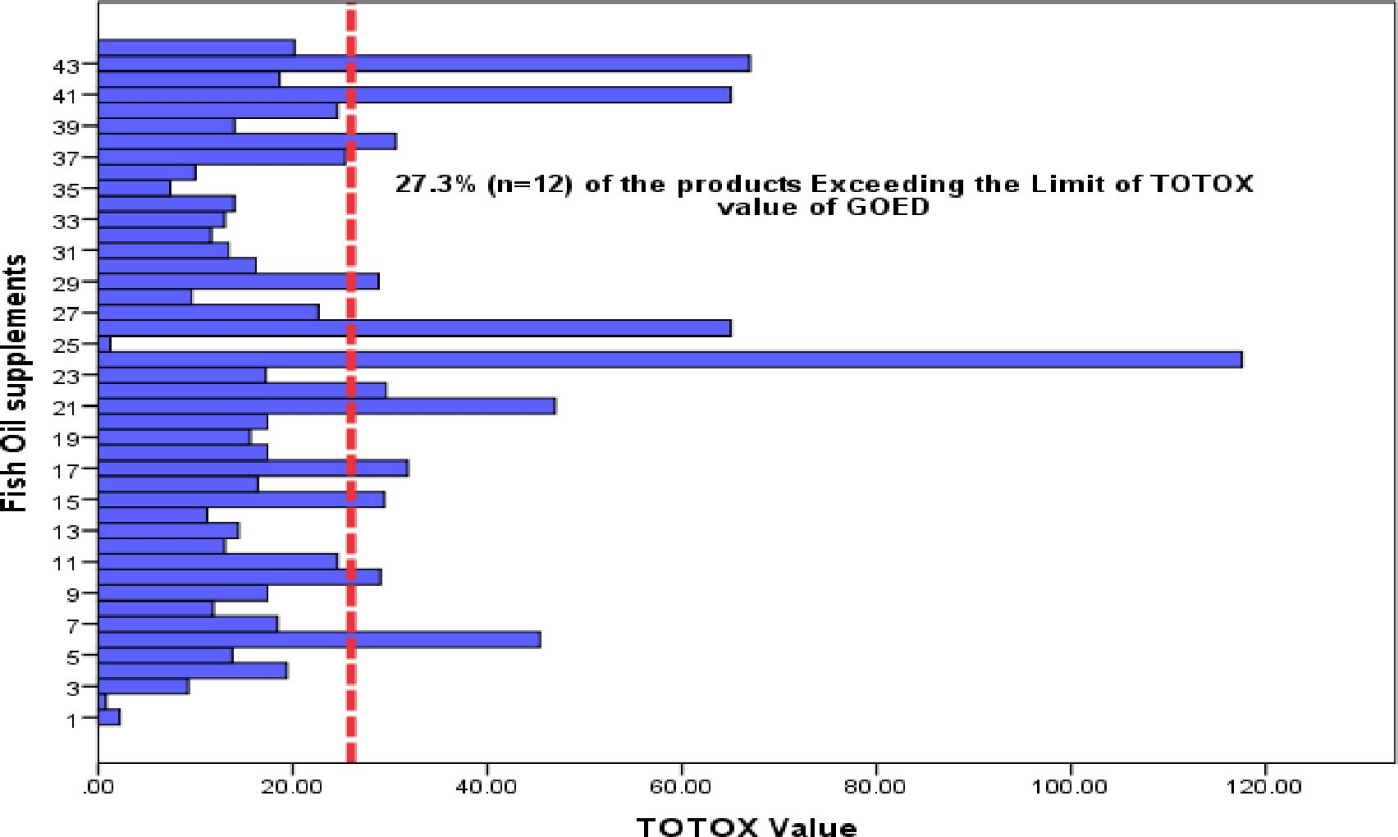
Bar chart of the estimated TOTOX for fish oil supplements (n = 44). The vertical dashed line is the maximum allowable limit of the TOTOX.

The estimate for the average PV value was 6.4 with a 95% CI [4.2–8.7] compared to the maximum allowable limit of 5 meq/kg. The estimate for the average P-AV value was 11 with a 95% CI [7.8–14.2] compared to the maximum allowable limit of 20. The estimate for the average TOTOX value was 23.8 meq/kg with a 95% CI [17.4–30.3] compared to the maximum allowable limit of 26 according to the GOED standards. Of the 44 tested fish oil supplements, 18 (40.9%) exceeded the recommended PV level (< 5 meq O2/kg), 3 (6.8%) exceeded the recommended P-AV level (< 20) and 12 (27.3%) exceeded the recommended TOTOX level (< 26) (Table 2). The estimated concentration of the oxidation parameters (PV, P-AV, and TOTOX) for the 44 products are presented graphically in histograms (Figs 1–3). The relevant maximum allowable limits are displayed as vertical “cut-off” limits. The results of the oxidative status (PV, P-AV, and TOTOX) stratified by sample characteristics of each sample are provided in Table 3.

**Table 3:** List of tested fish oil supplements according to oxidation parameters PV, P-AV and TOTOX values.

Sample code	Country of origin	Packaging type	Packaging colour	Dosage form	Vitamin E	PV value	P-AV value	TOTOX
**1**	Germany	Plastic	Aluminium strips	Chewable	No	1.1	1	2.2
**2**	Germany	Plastic	Aluminium strips	Chewable	No	0.4	1	0.8
**3**	USA	Plastic	Aluminium strips	Soft gel	Yes	2.1	5	9.2
**4**	USA	Plastic	Aluminium strips	Soft gel	Yes	3.7	12	19.4
**5**	USA	Plastic	Aluminium strips	Soft gel	Yes	3.9	6	13.8
**6**	Germany	Plastic	Aluminium strips	Capsules	Yes	20.2	5	45.4
**7**	India	Glass	Amber coloured	Syrup	No	7.7	3	18.4
**8**	Spain	Glass	Amber coloured	Syrup	Yes	5.9	1	11.8
**9**	Spain	Plastic	Aluminium strips	Capsules	No	7.7	2	17.4
**10**	USA	Plastic	Amber coloured	Capsules	Yes	9.5	10	29
**11**	USA	Plastic	Amber coloured	Capsules	No	9.3	6	24.6
**12**	Canada	Plastic	Transparent	Soft gel	No	3	7	13
**13**	Canada	Plastic	Transparent	Soft gel	No	2.2	10	14.4
**14**	Canada	Plastic	Transparent	Soft gel	No	2.1	7	11.2
**15**	Canada	Plastic	Transparent	Soft gel	No	5.7	18	29.4
**16**	Canada	Plastic	Transparent	Soft gel	No	3.2	10	16.4
**17**	Canada	Plastic	Transparent	Soft gel	No	8.9	14	31.8
**18**	Canada	Plastic	Transparent	Soft gel	No	3.7	10	17.4
**19**	Canada	Plastic	Transparent	Soft gel	No	3.3	9	15.6
**20**	Canada	Plastic	Transparent	Soft gel	No	4.2	9	17.4
**21**	Canada	Plastic	Transparent	Capsules	No	14.5	18	47
**22**	Canada	Plastic	Transparent	Soft gel	No	6.8	16	29.6
**23**	Canada	Plastic	Transparent	Capsules	No	5.1	7	17.2
**24**	Italy	Plastic	Aluminium strips	Capsules	No	40.3	37	117.6
**25**	USA	Plastic	Transparent	Chewable	No	0.6	1	1.2
**26**	Spain	Plastic	Aluminium strips	Capsules	Yes	27.5	10	65
**27**	Spain	Plastic	Aluminium strips	Capsules	Yes	8.8	5	22.6
**28**	UK	Plastic	Transparent	Capsules	Yes	1.8	6	9.6
**29**	UK	Plastic	Transparent	Capsules	No	5.9	17	28.8
**30**	UK	Plastic	Transparent	Capsules	No	2.1	12	16.2
**31**	UK	Plastic	Transparent	Capsules	Yes	2.2	9	13.4
**32**	UK	Plastic	Transparent	Capsules	No	1.8	8	11.6
**33**	USA	Plastic	Transparent	Capsules	No	4	5	13
**34**	USA	Plastic	Amber coloured	Capsules	No	2	10	14
**35**	France	Plastic	Amber coloured	Syrup	Yes	0.2	7	7.4
**36**	India	Plastic	Transparent	Capsules	No	3	4	10
**37**	India	Plastic	Transparent	Capsules	Yes	4.7	16	25.4
**38**	India	Plastic	Transparent	Capsules	Yes	7.8	15	30.6
**39**	India	Plastic	Transparent	Capsules	No	2	10	14
**40**	New Zealand	Plastic	Transparent	Capsules	No	4.3	16	24.6
**41**	India	Plastic	Transparent	Capsules	No	16	33	65
**42**	Germany	Glass	Amber coloured	Syrup	Yes	3.3	12	18.6
**43**	Germany	Glass	Amber coloured	Syrup	Yes	3.5	60	67
**44**	USA	Plastic	Amber coloured	Soft gel	No	7.6	5	20.2

### Comparison of oxidative parameters according to sample characteristics

Table 4 presents the distribution of oxidative parameters (PV, P-AV, and TOTOX) according to sample characteristics. The table also provides the estimates along with p-values based on the Mann-Whitney U test and Kruskal-Wallis test. Dosage forms showed statistically significant associations with the levels of oxidative status of the fish oil supplements. Capsules showed higher levels of PV and TOTOX compared to other dosage forms (P = 0.023 and P = 0.023, respectively), whereas syrup showed higher levels of P-AV than others (P = 0.025). There was also a statistical trend associating the levels of oxidation and packaging colour. Aluminium strips showed higher levels of PV, while amber coloured showed higher levels of P-AV (P = 0.042 and P = 0.052, respectively).

**Table 4:** Comparison of oxidative parameters according to sample characteristics.

		Peroxide value				Anisidine value				TOTOX value			
	Groups	Mean	Median	± SD	P-value	Mean	Median	± SD	P-value	Mean	Median	± SD	P- value
**Country of origin**	**USA**	**4.74**	**3.90**	**3.27**	**0.856**	**6.67**	**6**	**3.39**	**0.214**	**16.04**	**14**	**8.3**	0.660
	**EU**	**8.06**	**3.5**	**11.04**		**12.3**	**8**	**15.04**		**28.2**	**17.4**	**30.1**	
	**India**	**6.87**	**6.2**	**5.06**		**13.5**	**12.5**	**10.97**		**27.2**	**21.9**	**19.9**	
	**Canada**	**5.23**	**3.95**	**3.53**		**11.3**	**10**	**4.16**		**21.7**	**17.3**	**10.5**	
**Dosage forms**	**Syrup**	**4.12**	**3.5**	**2.8**	**0.023**	**16.6**	**7**	**24.6**	**0.040**	**24.6**	**18.4**	**24.1**	**0.025**
	**Soft gel**	**4.31**	**3.7**	**2.1**		**9.86**	**9.5**	**3.98**		**18.48**	**16.9**	**7.1**	
	**Chewable**	**0.70**	**0.60**	**0.36**		**1.0**	**----**	**----**		**1.40**	**1.20**	**0.72**	
	**Capsules**	**9.11**	**5.5**	**9.6**		**11.86**	**10**	**8.7**		**30.1**	**23.6**	**25.4**	
**Packaging colour**	**Transparent**	**4.76**	**3.7**	**3.7**	**0.042**	**11.48**	**10**	**6.4**	**0.052**	**20.95**	**16.4**	**13.4**	**0.433**
	**Aluminium strips**	**11.57**	**5.8**	**13.4**		**8.40**	**5**	**10.6**		**31.34**	**18.4**	**36.2**	
	**Amber coloured**	**5.44**	**5.9**	**3.3**		**12.67**	**7**	**18.1**		**23.44**	**18.6**	**17.6**	
**Vitamin E**	**Yes**	**7.0**	**3.9**	**7.4**	**0.603**	**11.9**	**9**	**13.9**	**0.684**	**25.9**	**19.4**	**19.1**	**0.569**
	**No**	**6.2**	**4**	**7.5**		**10.6**	**9**	**8.5**		**22.8**	**17.2**	**22.4**	
**Pure fish oil (omega-3 fatty acid**)	**Yes**	**6.13**	**3.50**	**6.3**	**0.832**	**10.3**	**9.5**	**8.2**	**0.603**	**22.4**	**16**	**26.3**	**0.747**
	**No**	**6.63**	**4.50**	**9.4**		**11.5**	**8.5**	**11.7**		**24.6**	**19.8**	**18.1**	

P-value reported above for comparisons between variable levels “categories-levels” using the Kruskal-Wallis test.

Table 5 presents the bivariate analysis correlation between the total concentration of omega-3 fatty acid and oxidative parameters. The results showed a significant relationship between total concentration of omega-3 fatty acid and PV value (r = 0.816, P< 0.001), AV value (r = 0.492, P = 0.002) and TOTOX value (r = 0.813, P< 0.001).

**Table 5:** Bivariate analysis between the oxidative parameters and total concentration of Omega-3 fatty acid.

Oxidative parameters		Total omega-3 fatty acid (mg)
Peroxide value	r	0.816
	P	< 0.001 *
Anisidine value	r	0.492
	P	0.002*
TOTOX value	r	0.813
	P	< 0.001 *

*Correlation is significant at the 0.05 level, r = correlation coefficient, the results obtained from the Pearson correlation test.

## Discussion

Fish oil supplements that are rich in n-3 PUFAs are among the most widely used dietary supplements globally, with millions of people consuming them regularly. There has always been public concern that these products should be guaranteed safe and of good quality, especially as these types of fish oil supplements are extremely susceptible to oxidative degradation. A number of studies have evaluated the oxidative quality of non-prescription fish oil supplements and found significant variations in the frequency of excess oxidation [34].

This research examined the oxidative status of 44 dietary supplements containing fish oil available for purchase in the UAE. Of the supplements tested, 40.9% of the products had PV levels above the recommended maximum (5 meq O2/kg), higher than the levels found in the USA (27%) [35] and in one New Zealand study (28%) [36], but these levels were lower than those found in South Africa [37] and another New Zealand study [38] where over 80% of products had PV levels exceeding the recommended maximum.

In addition, of the 44 dietary supplements containing fish oil that were examined, 6.8% had levels above the maximum recommended for AV; this was well below the AV values found in the two New Zealand studies referenced (14% [36] and 25% [38], respectively).

Furthermore, 27.3% of the 44 products tested exceeded the recommended TOTOX threshold; this was below the values found in Canada (39%) [24] or either New Zealand study (23% [36] and 23% [38], respectively).

Overall, our results demonstrated that there is a satisfactory and reassuring level of adherence to the oxidative quality parameters for dietary supplements containing fish oil; this may be attributed to the UAE's regulatory system, in which health regulators and municipalities require that all dietary supplements containing fish oil be registered to ensure that they are safe, efficacious, and of good quality before being sold in the UAE. The outcomes of this research are in accord with the findings of other researchers [39–41].

However, other research has reported far greater incidences of non-compliance, which may be anomalies. It is suggested that more care should be taken to avoid PUFA-rich oils becoming inadvertently oxygenated when the samples are being prepared; there should be greater awareness of how colorimetric assays can be affected by interference, and analytical methodologies and reporting should be improved so that quantitative assessments of the products in question will be more accurate [36].

Interestingly, when this type of supplement was available in capsule form, it was more likely to have high levels of PV and TOTOX compared to other forms of delivery. Additionally, supplements offered in syrup form showed higher levels of P-AV, those that were packaged in aluminium strips had higher levels of PV, and those in amber-coloured strips had higher levels of P-AV.

These outcomes may have been caused by problems with quality assurance and the lack of a standard method of obtaining and processing raw materials, meaning that different batches of these supplements can vary considerably, making it more complex to analyse their safety and efficiency. This makes it extremely important that companies that produce fish oil and those that produce the final product use reliable testing methodologies to create specifications and claims for their products and that analyses use accredited laboratories that have a provable track record of accuracy [36].

### Study limitations

This study has several limitations that should be considered. First, power analysis and sample size estimation were not computed because there is limited research investigating the oxidation status of fish oil supplements in the UAE. Second, this study did not report the fish oil sources, whether from the same fish type or from variable sources such as krill oil, salmon oil and others. Third, this study found large standard deviations for the peroxide markers, which can be attributed to the adulteration of fish oil supplement products by the manufacturers. For example, some manufacturers may add omega-3 fatty acids in unnatural forms, which could be more susceptible to degradation. Thus, a system to inspect and license manufacturing plants should be implemented. Moreover, laboratory-based analysis procedures that are governed by an accredited laboratory that employs a standard methodology should be implemented. Fourth, the current study did not assess the complete fatty-acid composition of the analysed fish oil supplement products; therefore, a total fatty-acid analysis by GC could help elucidate the fatty-acid composition of the products, while FT-IR and LC-MS/MS could determine the sources of LCPUFAs in each product.

## Conclusions

This research shows that the majority of, although not all, the fish oil supplements tested are compliant with GOED oxidative quality standards. Nevertheless, it is clear that there should be a high level of inspection and control regarding authenticity, purity, quality, and safety in the process of production and supply of dietary supplements containing fish oils.

## Supporting information

S1 Data (XLSX)
